# Energy Budget, Behavior and Leptin in Striped Hamsters Subjected to Food Restriction and Refeeding

**DOI:** 10.1371/journal.pone.0054244

**Published:** 2013-01-23

**Authors:** Zhi-Jun Zhao, Qiao-Xia Zhu, Ke-Xin Chen, Yu-Kun Wang, Jing Cao

**Affiliations:** 1 School of Life and Environmental Sciences, Wenzhou University, Wenzhou, Zhejiang, China; 2 School of Agricultural Science, Liaocheng University, Liaocheng, Shandong, China; CIMA. University of Navarra, Spain

## Abstract

Food restriction induces a loss of body mass that is often followed by rapid regaining of the lost weight when the restriction ends, consequently increasing a risk of development of obesity. To determine the physiological and behavioral mechanisms underlining the regaining, striped hamsters were restricted to 85% of initial food intake for 4 weeks and refed *ad libitum* for another 4 weeks. Changes in body mass, energy budget, activity, body composition and serum leptin level were measured. Body mass, body fat mass and serum leptin level significantly decreased in food-restricted hamsters, and increased when the restriction ended, showing a short “compensatory growth” rather than over-weight or obesity compared with *ad libitum* controls. During restriction, the time spent on activity increased significantly, which was opposite to the changes in serum leptin level. Food intake increased shortly during refeeding, which perhaps contributed to the rapid regaining of body mass. No correlation was observed between serum leptin and energy intake, while negative correlations were found in hamsters that were refed for 7 and 28 days. Exogenous leptin significantly decreased the time spent on activity during food restriction and attenuated the increase in food intake during refeeding. This suggests that low leptin in restricted animals may function as a starvation signal to induce an increase in activity behavior, and high leptin likely serves as a satiety signal to prevent activity during refeeding. Leptin may play a crucial role in controlling food intake when the restriction ends, and consequently preventing overweight.

## Introduction

Periods of restricted food intake induce a loss of body mass that is often followed by rapid regaining of the lost weight when the restriction ends, during which physiological regulations associated with either energy intake or expenditure, or the both are reported to be involved [1]–[8]. However, the results related to energy budget and behaviors in response to food restriction and refeeding remain controversial. For example, the energy spent for the rate of resting metabolism (RMR) and activity behavior decreased in food-restricted laboratory mice and rats [7], [9], [10]. In contrary, Siberian hamsters (*Phodopus sungorus*) and other hamster species increased activity associated foraging and food hoarding behaviors in response to food shortage [11]–[15]. During refeeding, laboratory rats regained body mass and fat mass, showing a “compensatory growth” [2], [3], [16]. Some wild rodents also showed “compensatory growth”, but to much less extent compared with that observed in laboratory animals [17]. This paradox may reflect different energy strategy and behavioral patterns in wild animals from that in laboratory rodents.

Leptin, the product of the *ob* gene, is mainly expressed in adipose tissue and plays important roles in the regulation of both energy intake and expenditure [18]–[20]. It was reported that serum leptin level reduced during food restriction and increased during refeeding [5], [6], [17]. Leptin administration to food-restricted laboratory rats reduced food intake and prevented the regain of body mass [20]. In addition, exogenous leptin inhibited food-deprivation-induced increases in food intake and food hoarding in Siberian hamsters [15]. These results make leptin to be a possible candidate involved in the regulations of energy budget and behavior in response to food restriction and refeeding in both laboratory and wild animals.

The striped hamster (*Cricetulus barabensis*) is a major rodent in northern China and is also distributed in Russia, Mongolia, and Korea [6]. The hamsters feed on stems and leaves of plant during summer and on foraging crop seeds in winter [6], [21]–[23]. Thus the species must experience great seasonal fluctuations in food quality and availability [6]. Whereas, unlike other wild rodents, such as Djungarian hamsters (*Phodopus sungorus*) [24], [25], Brandt's voles (*Lasiopodomys brandtii*) [26] and Mongolian gerbils (*Meriones unguiculatus*) [27], striped hamsters do not show significant changes in body masses after being maintained in an outside enclosure over a year (Zhao ZJ, unpublished data). We previously found a significant decrease in body mass in stochastic food-restricted hamsters, followed by a slower regaining of body mass during refeeding than that in Swiss mice [28]. It suggests that striped hamster, showing different patterns of body mass regulation from both lab mice and other wild rodents, may become a potential model that is suitable for studying the resistance to over-weight when food restriction ends. In the present study, energy budget and activity behavior were measured in striped hamsters subjected to a successive food restriction for four weeks and refeeding for another four weeks. The effect of leptin supplement on energy budget and activity behavior was examined during both food restriction and refeeding. We hypothesized that regulations of energy budget and activity behavior would be employed to cope with the changes in food availability, but failing to regain the lost weight when the restriction ended. Leptin might be involved in changes in energy budget and activity, and consequently played a key role in the resistance to over-weight in striped hamsters experiencing food restriction and refeeding.

## Materials and Methods

### Ethics Statement

This study was in compliance with the Animal Care and Use Committee of Liaocheng University. The experiment procedure and protocol were approved by the Committee (Permit Number: 11-0219-011).

### Animals and experiment protocol

Striped hamsters were obtained from a laboratory-breeding colony started with animals that were initially trapped from farmland at the center of Hebei province (115°13′E, 38°12′S), North China Plain. Environmental temperature was kept constant at 21±1°C with a 12 h∶ 12 h light∶ dark cycle (lights on at 0800 h). Food (standard rodent chow; produced by Beijing KeAo Feed Co.) and water were provided *ad libitum*. The macronutrient composition of the diet was 6.2% crude fat, 20.8% crude protein, 23.1% neutral detergent fiber, 12.5% acid detergent fiber, and 10.0% ash, and the caloric value is 17.5 kJ/g. Adult male hamsters, 4–5 months old, were singly housed in plastic cages (29×18×16 cm) with fresh saw dust bedding for two weeks before the experiments.

#### Experiment 1: Effects of food restriction (FR) and refeeding (Re) on body mass and food intake

Twenty four male hamsters were assigned randomly into either control group (Con, *n* = 12) that animals were fed *ad libitum* for 8 weeks, or FR and Re group (FR-Re, *n* = 12) in which each hamster was restricted to 85% of initial food intake for 28 days and refed *ad libitum* for another 28-days. Body mass was measured every three days and food intake was determined on a daily basis. Before animals were restricted, food intake was calculated as the mass of food missing from the hopper every day, subtracting orts mixed in the bedding. Prior to the initiation of food restriction, initial food intake for each animal was calculated as the average of daily food intake over 7 days. Each hamster in FR-Re group was provided with 85% of initial food intake only during FR period, making food-restricted hamster had a 15% reduction of caloric intake. Food was given the same time each day at 1900 h following body mass measurements.

#### Experiment 2: Effects of FR and Re on behavior, energy budget, body composition

Fifty six hamsters were assigned randomly into one of the following 7 groups (*n* = 8 in each group): controls that were fed *ad libitum* for 8 weeks; FR- d 1, FR- d 7 and FR- d 28 groups, animals were restricted to 85% of initial food intake for 1, 7 and 28 days, respectively; and Re-d 1, Re-d 7 and Re-d 28 groups, during which animals were restricted to 85% of initial food intake for 4 weeks and were then refed *ad libitum* for 1, 7 and 28 days, respectively. At the end of the experiment, behavior observation was made, and RMR and energy budget were measured.

### Behavior observation

Behavior observations were made in 4 hamsters from each group over a day (24 h). Observations were performed using computer-connected infrared monitors (SONY, 420 TV line) and were automatically stored in computer, which were then subjected to operator analysis. General activity included any active movement such as walking around the cage and climbing on the cage bars [29], [30]. The time spent on activity was recorded and expressed as min/h and min/24 h, respectively.

### RMR

RMR was quantified as the rate of oxygen consumption, using a computerized open-flow respirometry system (Sable system, USA). Air was pumped at a rate of 750–850 ml/min through a cylindrical sealed Perspex chamber at 29±0.5°C (within the thermal neutral zone of this species, [23], [31]. Gases leaving the chamber were dried (silica gel) and sampled using an oxygen analyzer at a flow rate of 150–175 ml/min. The data were averaged and collected every 10 s by a computer connected analogue-to-digital converter (STD-UI2, Sable system), and analyzed using a standard software (Sable system). RMR was measured for 2.5 hours between 11: 00 and 17: 00, and calculated from the lowest rate of oxygen consumption over 5 min, using the equation: VO_2_ = Flow rate×(FiO_2_−FeO_2_)/(1−FiO_2_×(1−RQ)), where FiO_2_ is input fractional concentration of O_2_ to the chamber; FeO_2_ is excurrent fractional concentration of O_2_ from the chamber; and RQ is respiratory quotient [32]. Here, RQ was assumed to be 0.85 [33], [34]. RMR was then corrected to the standard temperature and air pressure (STP) conditions.

### Energy budget

Food was provided quantitatively, and the spillage of food mixed with bedding and feces were collected from each cage over the last 2 days in control, FR- d 7, FR- d 28, as well as Re-d 7 and Re-d 28 groups, but over one day in FR- d 1 and Re-d 1 groups. The spillage of food and feces were sorted and separated manually after they were dried at 60°C to constant mass. Gross energy contents of the diet and feces were determined using a Parr 1281 oxygen bomb calorimeter (Parr Instrument, Moline, IL, USA). Gross energy intake (GEI), digestive energy intake (DEI), and apparent energy assimilation efficiency (digestibility) were calculated as follows [35]–[38]:

GEI (kJ·d^−1^) = food intake (g·d^−1^)×dry matter content of the diet (%)×energy content of food (kJ·g^−1^);

DEI (kJ·d^−1^) = GEI−(dry mass of feces (g·d^−1^)×energy content of feces (kJ·g^−1^));

Digestibility (%) = DEI/GEI×100%.

### Serum leptin levels

Animals were euthanized by decapitation between 0900 and 1100 h on the day next to RMR measurements. Trunk blood was collected for serum leptin measurements. Serum leptin level was quantified by radio-immunoassay (RIA) using the Linco ^125^I Multi-species Kit (Cat. No. XL-85K, Linco Research Inc.), following the standard kit instructions. The lower and upper limits of the assay kit were 1 and 50 ng/ml, and the inter- and intra-assay variations were <3.6% and 8.7%, respectively.

### Body composition

After trunk blood was collected, the gastrointestinal tracts were separated, and liver, heart, lung, spleen pancreas and kidneys were also removed. The remaining carcass (including the brain, but excluding the thyroid and urinary bladder) was weighed (to 0.001 g) to determine wet mass, dried in an oven at 60°C for 10 days to a constant mass, and then weighed (to 0.001 g) again to determine dry mass. Total body fat was extracted from the dried carcass by ether extraction in a Soxhlet apparatus [37], [38].

#### Experiment 3: Effect of leptin supplement on food intake and behavior during FR and Re

Sixteen hamsters were randomly assigned into one of the four groups: Ad-PBS, hamsters that were fed *ad libitum* and treated with PBS; Ad-leptin, Ad hamsters that were treated with leptin; FR-PBS, FR hamsters that were treated with PBS; FR-leptin, FR hamsters that were treated with leptin. Animals were fed *ad libitum* for 14 days in Ad groups. FR hamsters were restricted to 85% of initial food intake for 10 days, and then refed *ad libitum* for 4 days. On day 8, hamsters were anesthetized with isoflurane and implanted subcutaneously on the dorsal side with a miniosmotic pump (Alzet model 1007D; capacity, 100 µl; release rate, 0.5 µl/h; duration, 7 days; Durect, Cupertino, CA) containing either recombinant murine leptin (100 µg dissolved in 100 µl phosphate-buffered saline [PBS], purchased from Peprotech, USA) or PBS. Body mass and food intake were measured daily according the method mentioned in experiment 1. Activity observation was performed as described in experiment 2 and the time spent on activity was recorded and expressed as min/24 h. Animals were euthanized by decapitation and trunk blood was collected for serum leptin measurements as the same methods mentioned in “Serum leptin levels”.

### Statistics

Data were expressed as the means ± SE and analyzed using SPSS 13.0 statistic software. Experiment 1, changes in body mass and food intake throughout FR and Re period were analyzed using repeated one-way ANOVA measurements, and differences between the two groups on any day points were examined using independent *t*-tests. Experiment 2, differences in activity behavior, RMR, energy budget, serum leptin levels and body composition between the seven groups were examined using one-way ANOVA or ANCOVA with body mass or carcass mass as a covariate, followed by Tukey's HSD post-hoc tests where appropriate. Experiment 3, body mass change throughout the experiment was examined using repeated measurements. Differences in body mass change, food intake and activity on any day points as well as serum leptin levels were examined using two-way ANOVA (FR×leptin), followed by Tukey's HSD post-hoc tests where required. Correlations of leptin with fat content and gross energy intake were examined using a Pearson correlation analyses. The level of significance was set at *P*<0.05.

## Results

### Effects of food restriction (FR) and refeeding (Re) on body mass and food intake

#### Food intake

**Figure 1:**
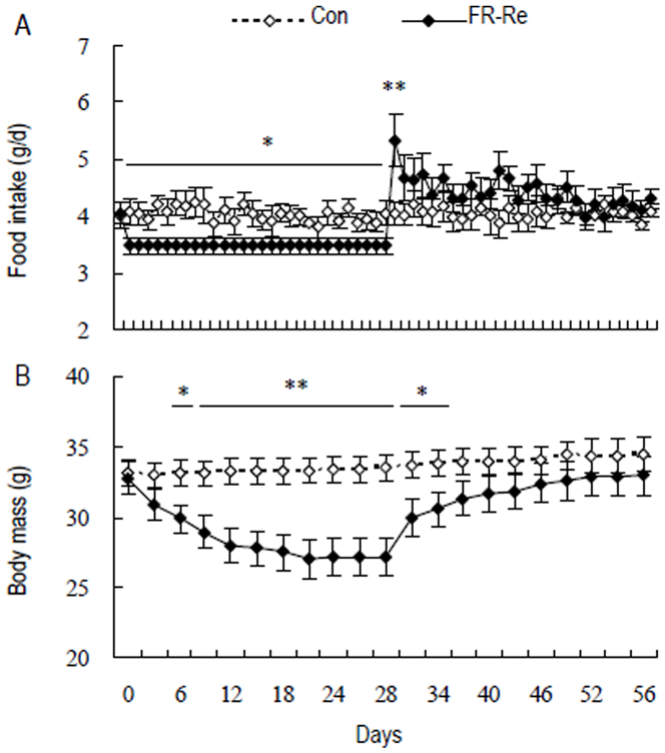
Food intake (A) and body mass (B) in striped hamsters. Con, hamsters that were fed *ad libitum*; FR-Re, hamsters that were restricted to 85% of initial food intake for 28 days and refed *ad libitum* for another 28 days. Values are means ± SE. Asterisks denote statistically significant differences between Con and FR-Re groups at the same period of time; *, *P*<0.05 and **, *P*<0.01.

Food intake was not different between Con and FR-Re groups prior to the experiment (d 0, *t*_21_ = 0.16, *P*>0.05, Fig. 1A). There were no changes in food intake throughout the experiment in Con group (d 1–56, *F*_55,605_ = 0.62, *P*>0.05), while significant changes were observed in FR-Re group (d 1–56, *F*_55,550_ = 13.81, *P*<0.01). During restriction, FR-Re animals were provided with 85% of initial food intake only, which was lower than that of control animals (d 1, *t*_21_ = 2.20, *P*<0.05, d 28, *t*_21_ = 2.22, *P*<0.05). During refeeding, FR-Re animals consumed more food than control animals (d 29, Con, 4.0±0.2 g/d, FR-Re, 5.3±0.5 g/d, *t*_21_ = 2.71, *P*<0.05), whereas food intake was not statistically different between the two groups on day 30 and thereafter (d 30, *t*_21_ = 1.53, *P*>0.05, d 56, *t*_21_ = 1.10, *P*>0.05, Fig. 1A).

#### Body mass

There was no difference in body mass between Con and FR-Re groups before the experiment started (d 0, *t*_21_ = 0.21, *P*>0.05, Fig. 1B). Control hamsters increased their weight from 33.1±0.9 g on day 0 to 34.4±1.2 g on day 56 (days 0–56, *F*_20,220_ = 6.75, *P*<0.05). Body mass significantly decreased in FR-Re animals during restriction, which decreased by 16% on day 18 compared with on day 0 (days 1–18, *F*_6,60_ = 41.52, *P*<0.01), and then lowered to a minimum of around 27 g between days 21 and 28. On the first few days of refeeding, body mass shortly increased in FR-Re groups (days 34–56, *F*_8,80_ = 7.40, *P*<0.01). FR-Re animals showed lower body mass than control animals on day 6 till day 34 (d 6, *t*_21_ = 2.39, *P*<0.05, d 34, *t*_21_ = 2.05, *P*<0.05). Body mass was not statistically different between the two groups on day 37 and thereafter (d 37, *t*_21_ = 1.63, *P*>0.05, d 56, *t*_21_ = 0.80, *P*>0.05, Fig. 1B).

### Effects of FR and Re on behavior, energy budget, body composition

#### Activity

**Figure 2:**
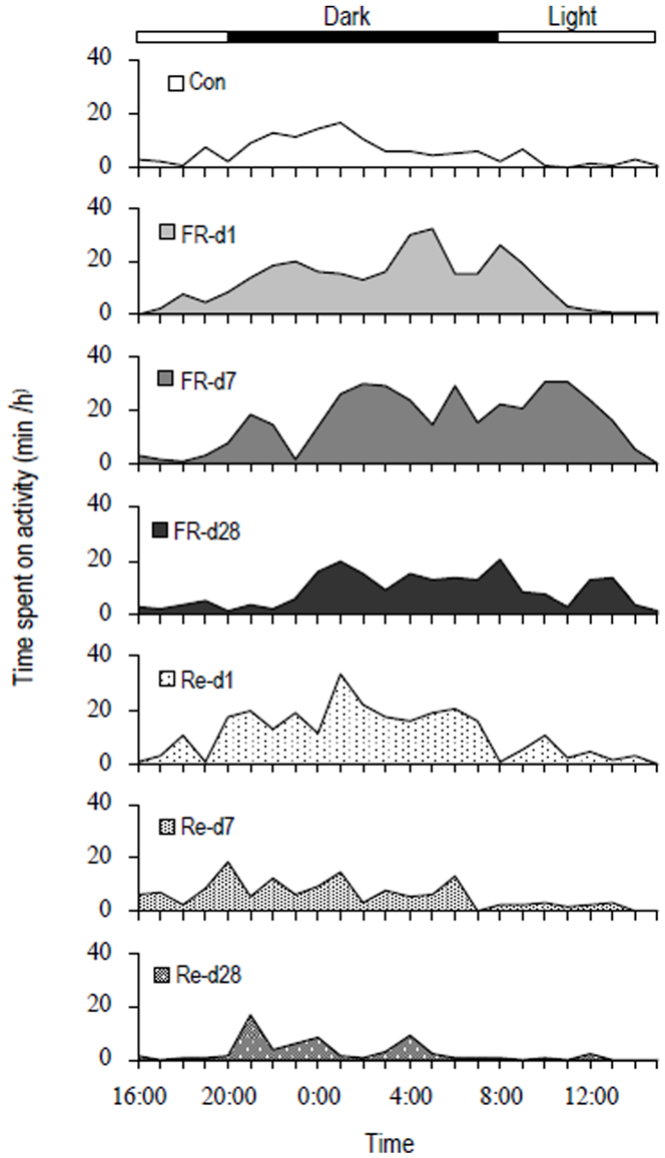
Activity patterns in striped hamsters. Con, hamsters that were fed *ad libitum*. FR-d 1, FR-d 7, FR-d 28, hamsters that were restricted to 85% of initial food intake for 1, 7 and 28 days; Re-d 1, Re-d 7 and Re-d 28, hamsters that were restricted to 85% of initial food intake for 28 days and refed *ad libitum* for 1, 7 and 28 days, respectively. Values are average from four animals in each group.

**Figure 3:**
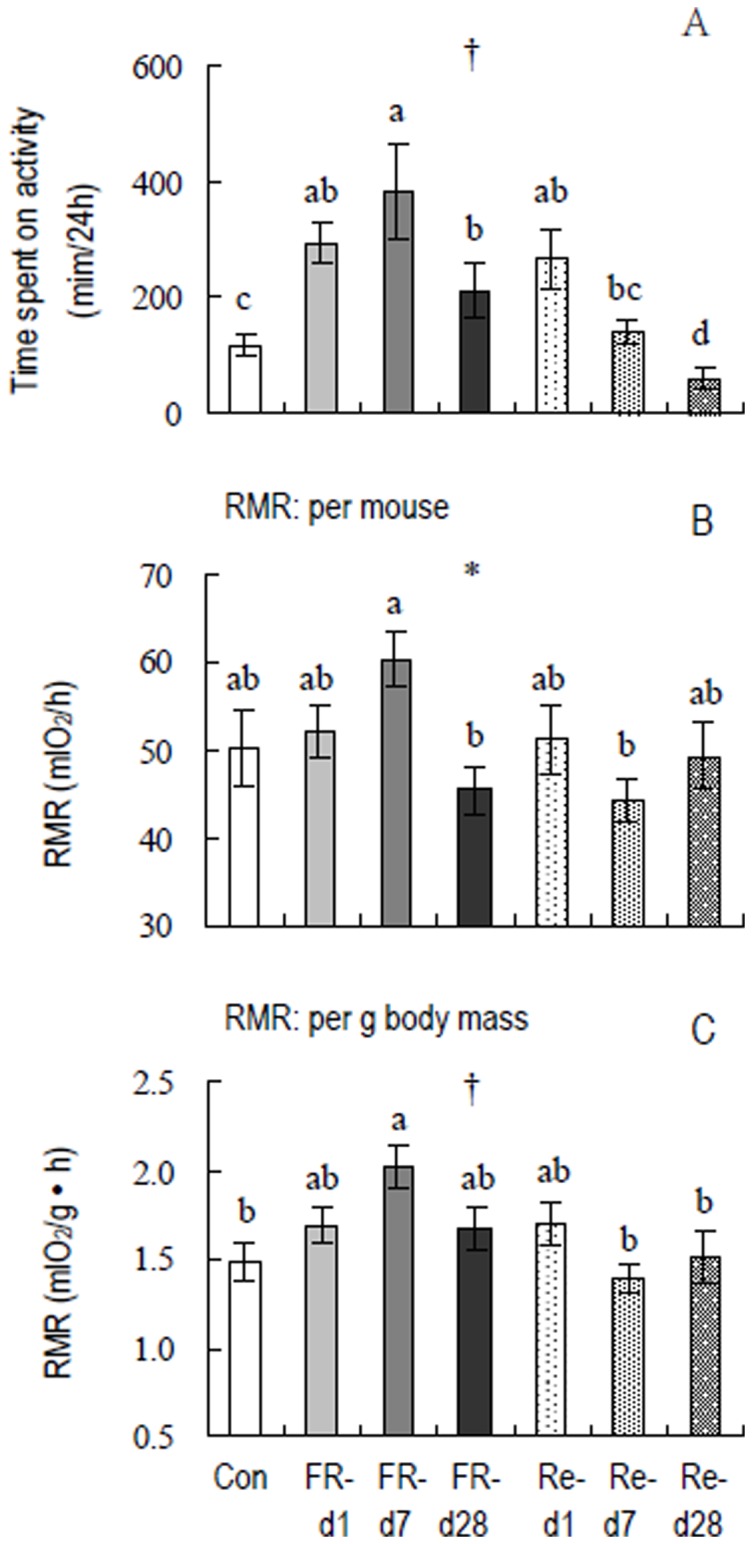
Time spent on activity (A) and resting metabolic rate (RMR) per mouse (B), and per g body mass (C), in striped hamsters subjected to food restriction and refeeding. Groups are the same as in Fig. 2. Effect of food restriction and refeeding is significant: *, *P*<0.05 and †, *P*<0.01. Different letters above the columns indicate significant differences between the seven groups (*P*<0.05).

Activity behavior usually occurred during the dark phase in control hamsters, while during the day phase they spent almost all the time on the rest (Fig. 2). During food restriction, FR-Re hamsters spent significantly more time on activity both during the dark and the light phase than controls. During refeeding, FR-Re hamsters still showed high activity behavior on day 1 (Re d 1), whereas they decreased the time spent on activity on day 7 (Re d 7) and thereafter. Activity behavior was affected by FR-Re (*F*_6,27_ = 6.27, *P*<0.01, Fig. 3A), by which FR-Re animals spent more time on activity during food restriction than controls (post Hoc, *P*<0.05). On day Re 28, the time spent activity was significantly less in FR-Re group than controls (Re d 28, post Hoc, *P*<0.05, Fig. 3A).

#### RMR

FR-Re had a significant effect on RMR when expressed either per mouse (mlO_2_/h, *F*_6,48_ = 2.53, *P*<0.05, Fig. 3B) or per gram body mass (mlO_2_/g • h, *F*_6,49_ = 3.28, *P*<0.01, Fig. 3C). RMR in FR-d 7 group was higher by 20% and 36% than controls when expressed per mouse and per weight, respectively (post hoc, *P*<0.05), while it was not statistically different between FR-d 28 group and controls (post hoc, *P*>0.05). During refeeding, RMR was significantly lower in Re-d 7 group than FR-d 7 group (post Hoc, *P*<0.05), whereas the differences between Re-d 1, Re-d 7, Re-d 28 groups and controls were not statistically different (post Hoc, *P*>0.05).

#### Energy budget

**Figure 4:**
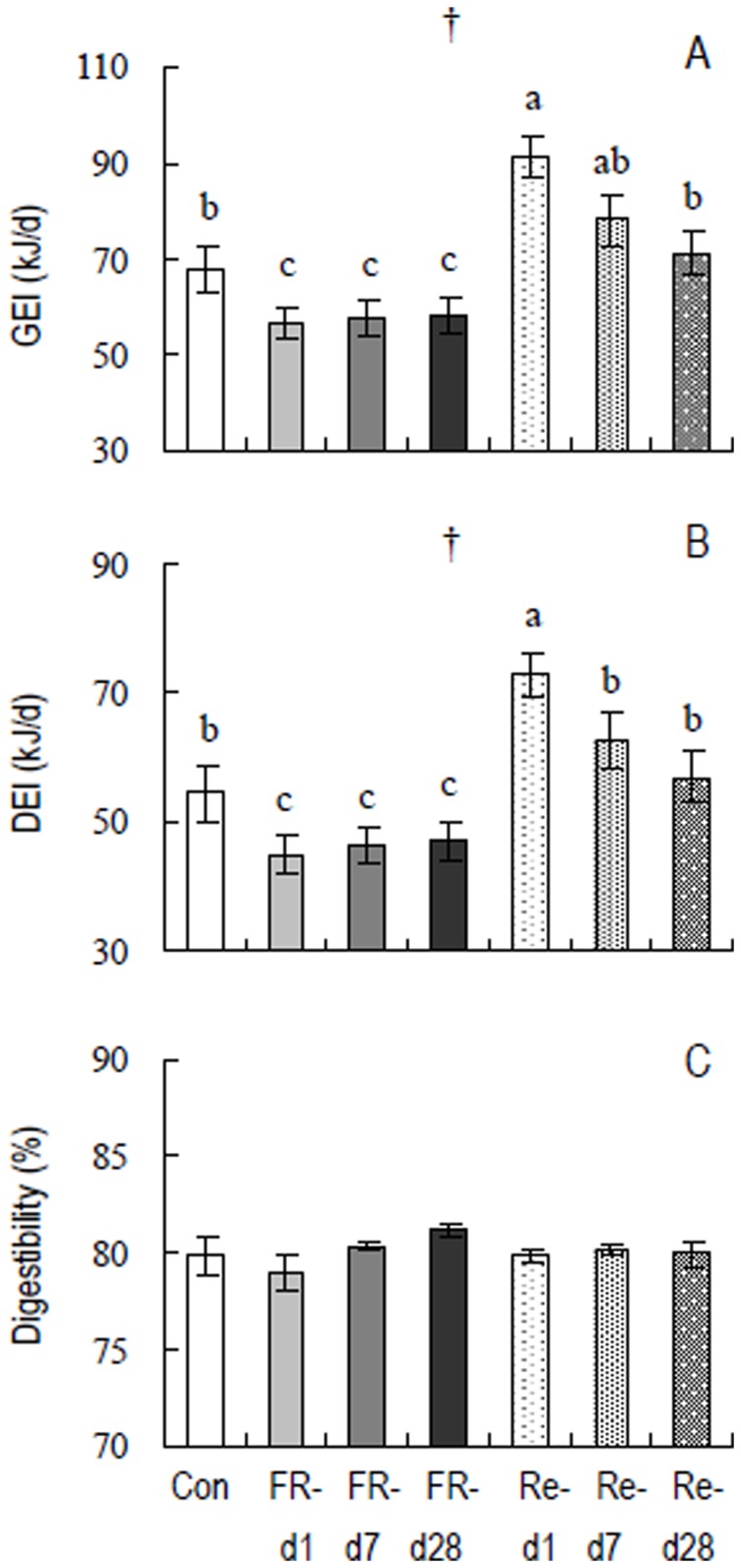
(A) Gross energy intake (GEI), (B) digestive energy intake (DEI), and (C) digestibility in striped hamsters subjected to food restriction and refeeding. Groups are the same as in Fig. 2. Effect of food restriction and refeeding is significant: †, *P*<0.01. Different letters above the columns indicate significant differences between the seven groups (*P*<0.05).

GEI was significantly affected by FR-Re (*F*_6,49_ = 8.95, *P*<0.01, Fig. 4A), FR-Re hamsters had lower GEI during restriction than controls (post Hoc, *P*<0.05). GEI was significantly higher in Re-d 1 group than control and FR-d 1, d 7 and d 28 groups (post Hoc, *P*<0.05), while it was not different between Re-d 7, Re-d 28 and control groups (post Hoc, *P*>0.05). DEI was similar to the changes observed in GEI, by which DEI was lower in FR-d 1, d 7 and d 28 groups, and higher in Re-d 1 group (*F*_6,49_ = 8.05, *P*<0.01, post Hoc, *P*<0.05, Fig. 4B). Digestibility was not affected by FR-Re, and no difference was observed between the 7 groups (*F*_6,49_ = 1.18, *P*>0.05, post Hoc, *P*>0.05, Fig. 4C).

#### Carcass mass and fat content

Wet and dry masses of carcass were significantly affected by FR-Re (Table 1), which were lower in FR-d 28 group than that in Con group (post Hoc, *P*<0.05). Fat mass and fat content were also affected by FR-Re. Fat mass and fat content were significantly lower in FR-d 28 groups than controls (post Hoc, *P*<0.05), while the difference between Con, Re-d 7 and Re-d 28 groups was not significant (post Hoc, P>0.05, Table 1).

**Table 1:** Masses of carcass and fat, fat content and serum leptin levels in striped hamsters subjected to food restriction and refeeding.

	Con	FR-d 1	FR-d 7	FR-d 28	Re-d 1	Re-d 7	Re-d 28	*P*
Body mass (g)	33.5±1.3^a^	31.2±1.3^ab^	30.3±1.2^ab^	27.6±1.0^b^	29.9±0.7^ab^	31.9±1.2^ab^	33.3±1.7^a^	*
Carcass								
Wet mass (g)	24.6±1.1^a^	23.1±0.8^ab^	22.0±0.9^ab^	20.3±0.4^b^	21.5±0.4^ab^	23.6±0.7^ab^	25.2±1.4^a^	**
Dry mass (g)	8.6±0.4^a^	8.0±0.4^ab^	7.5±0.3^ab^	6.9±0.2^b^	7.4±0.3^ab^	8.3±0.3^ab^	8.6±0.5^a^	**
Fat mass (g)	2.7±0.2^a^	2.2±0.3^ab^	1.6±0.2^bc^	1.2±0.1^c^	1.6±0.1^bc^	2.6±0.3^a^	2.5±0.2^a^	**

Con, hamsters that were fed *ad libitum*. FR-d 1, FR-d 7, FR-d 28, hamsters that were restricted to 85% of initial food intake for 1, 7 and 28 days; Re-d 1, Re-d 7 and Re-d 28, hamsters that were restricted to 85% of initial food intake for 28 days and refed *ad libitum* for 1, 7 and 28 days, respectively. Values are means ± SE.

* , Significant differences between means (*P*<0.05),

** , *P*<0.01.

#### Serum leptin

**Figure 5:**
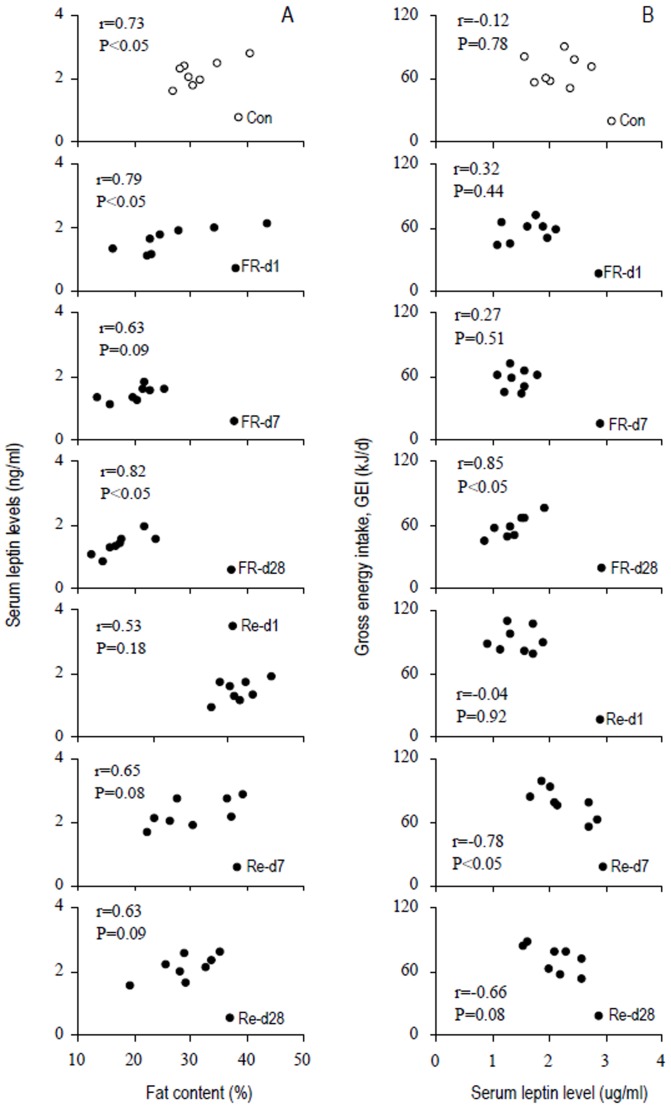
(A) Correlation between serum leptin levels and fat content and (B) correlation between gross energy intake (GEI) and fat content in striped hamsters subjected to food restriction and refeeding. Groups are the same as in Fig. 2. Data are plotted.

Serum leptin level was significantly affected by FR-Re, which was significantly lower in FR-d 1, d 7 and d 28 groups than controls (Table 1). Serum Leptin was still lower in Re-d 1 group compared with controls, but it increased significantly in Re-d 7 and Re-d 28 groups, which were similar to that observed in control group (Table 1). There was a positive correlation between serum leptin and fat content in controls, this correlation was also observed in other six groups (Fig. 5A). No correlation was observed between serum leptin and GEI in control hamsters (Fig. 5B). Serum leptin was positively correlated with GEI in FR-d 28 group, but no correlations were found in FR-d 1 and FR-d 7 groups. Hamsters in Re-d 7 and Re-d 28 groups showed significantly negative correlations between serum leptin and GEI in (Fig. 5B).

### Effect of leptin supplement on food intake and behavior during FR and Re

#### Body mass change

**Figure 6:**
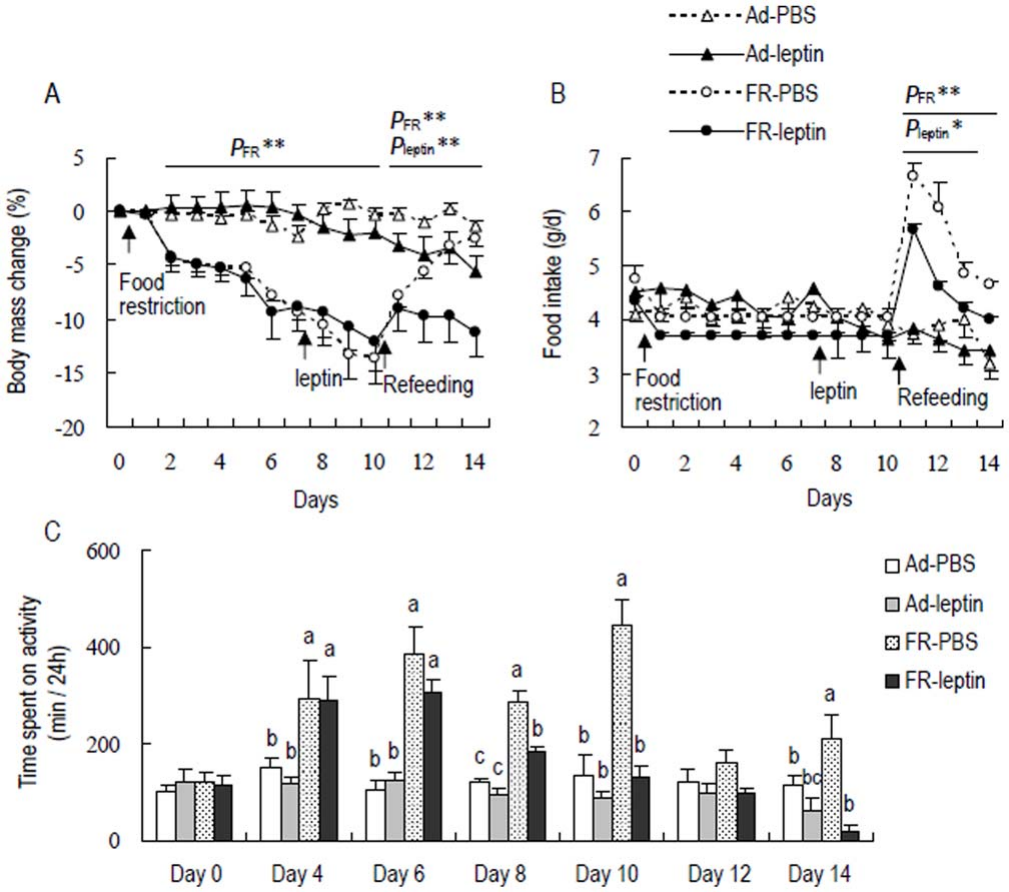
Effects of leptin administration on body mass change (A), food intake (B) and the time spent on activity (C) in striped hamsters. Ad-PBS, hamsters that were fed *ad libitum* and treated with PBS; Ad-leptin, Ad hamsters that were treated with leptin; FR-PBS, food-restricted (FR) hamsters that were treated with PBS; FR-leptin, FR hamsters that were treated with leptin. *P*_FR_ **, significant effect of FR (*P*<0.01); *P*_leptin_ *, significant effect of leptin manipulation (*P*<0.05), **, *P*<0.01; Different letters above the columns indicate significant differences between the groups (*P*<0.05).

Body mass was not different between the four groups prior to the experiment (d 0, FR, *F*_1,12_ = 0.84, *P*>0.05; leptin, *F*_1,12_ = 0.01, *P*>0.05, Fig. 6A). Food restriction had a significant effect on body mass change on day 1 till day 10, and restricted hamsters showed lower body mass than Ad animals (d 1, *F*_1,12_ = 12.25, *P*<0.01, d 10, *F*_1,12_ = 34.87, *P*<0.01). Leptin supplement had no effect on body mass change during food restriction (d 8, *F*_1,12_ = 0.03, *P*>0.05, d 10, *F*_1,12_ = 0.01, *P*>0.05), while had a significant impact on body mass change during refeeding (d 12, *F*_1,12_ = 5.62, *P*<0.01, d 14, *F*_1,12_ = 20.84, *P*<0.01). During refeeding phase, body mass increased from −13.6±2.4% on day 10 to −2.5±0.7% on day 14 in FR-PBS group (d 10–14, *F*_4,12_ = 8.43, *P*<0.01), while it did not change in FR-leptin group between these days (d 10, −12.1±2.7%, d 14, −11.2±2.3%, d 10–14, post hoc, *P*>0.05, Fig. 6A).

#### Effect of leptin administration on food intake

Food intake did not differ between the four groups prior to the initiation of food restriction (d 0, FR, *F*_1,12_ = 0.82, *P*>0.05; leptin, *F*_1,12_ = 0.02, *P*>0.05, Fig. 6B). During food restriction, food-restricted hamsters consumed 15% less food than *ad libitum* animals (d 1, *F*_1,12_ = 3.52, *P* = 0.09). 0n day 8 till 10, leptin supplement did not affect food intake in either *ad libitum* or food-restricted hamsters (d 8, *F*_1,12_ = 0.18, *P*>0.05; d 10, *F*_1,12_ = 1.48, *P*>0.05). During refeeding phase, food intake was higher in FR-PBS hamsters than Ad-PBS hamsters (FR, d 11, *F*_1,12_ = 146.12, *P*<0.01, d 14, *F*_1,12_ = 18.78, *P*<0.01, Fig. 6B). Leptin supplement had a significant effect on food intake on day 11 till 13 (d11, *F*_1,12_ = 4.91, *P*<0.05), by which food intake increased by 79.1%, 55.8% and 21.3% on day 11, 12 and 13 in FR-PBS group relative to Ad-PBS group, respectively, but elevated by only 52.7%, 18.6% and 5.6% in FR-leptin group (post hoc, *P*<0.05). However, the significant effect of leptin supplement on food intake disappeared on day 14 (*F*_1,12_ = 0.72, *P*>0.05). No difference in food intake was observed between Ad-PBS and Ad-leptin groups (post hoc, *P*>0.05, Fig. 6B).

#### Effect of leptin administration on activity

There was no group difference in time spent on activity on day 0 (FR, *F*_1,12_ = 0.11, *P*>0.05; leptin, *F*_1,12_ = 0.08, *P*>0.05, Fig. 6C). The time spent on activity was significantly affected by food restriction on day 4 till 10, and restricted animals spent more time on activity than Ad animals (d 4, *F*_1,12_ = 11.65, *P* = 0.01, post hoc, *P*<0.05; d 10, *F*_1,12_ = 23.24, *P*<0.01, post hoc, *P*<0.05). During refeeding, effect of restriction on activity was not significant on day 12 (*F*_1,12_ = 0.74, *P*>0.05) and day 14 (*F*_1,12_ = 0.94, *P*>0.05). Leptin supplement resulted in a significant reduction in activity, and hamsters spent 71% and 91% less time on activity in FR-leptin group on day 10 and day 14, respectively, than in FR-PBS group (d 10, *F*_1,12_ = 24.50, *P*<0.01, post hoc, *P*<0.05; d 14, *F*_1,12_ = 16.96, *P*<0.01, post hoc, *P*<0.05). The time spent on activity decreased by 36% and 45% in Ad-leptin than Ad-PBS groups on day 10 and 14, respectively, while the difference was not statistically different (d 10, post hoc, *P*>0.05, d 14, post hoc, *P*>0.05, Fig. 6C).

#### Effect of leptin administration on serum leptin

Serum leptin levels averaged 2.37±0.34 and 3.97±0.49 ng/ml in Ad-PBS and Ad-leptin groups, and 1.95±0.17 and 4.20±0.60 ng/ml in FR-PBS and FR-leptin groups, respectively. No effect of food restriction and refeeding on serum leptin was observed on the day following a 4-day's refeeding (*F*_1,12_ = 0.05, *P*>0.05). Leptin supplement resulted in significant increases in serum leptin for both hamsters fed *ad libitum* and hamsters under food restriction and refeeding (*F*_1,12_ = 19.90, *P*<0.001).

## Discussion

The change in food availability has been found to affect body mass in small mammals [17], [28], [39]–[41]. In the present study, we observed significant reductions in body mass, carcass mass, and body fat content in striped hamsters restricted to 85% of initial food intake. Weight losses were also observed in food-restricted C57/B6 mice [42], Swiss mice [28], [40], golden spine mice (*Acomys russatus, Muridae*) [17], [43] and Mongolian gerbils [6]. Inconsistently, body mass did not decrease in MF1 mice restricted to 80% of *ad libitum* food intake [10], and rats restricted to 75% of initial food intake [39]. The inconsistency may partly due to the different extent of restriction between the different studies above, since animals under severe food restriction often lose more weight than animals at softer restriction [28], [39]. Here, striped hamster lost weight more rapidly and significantly after restricted to 85% of initial food intake than either laboratory mice or rats, or other field rodents [10], [17], [42], [43]. This may suggest that striped hamsters, showing seasonal foraging behavior, are more sensitive to food shortage than the animals mentioned above. After being refed *ad libitum*, striped hamsters showed rapidly regaining of lost weight, showing “compensatory growth”, whereas the regaining was less and not followed by overweight compared with controls. Laboratory rats subjected to FR-Re, however, showed not only “compensatory growth” but also fatter than *ad libitum* controls [44]. The inconsistent results may be due to the species-specific energy budget strategy in response to the change of food availability [10].

In the present study striped hamsters consumed less food during food restriction than controls. When given free access to unlimited diet, they increased food intake by 33% compared with their counterpart controls (*P*<0.05). However, this increase was observed only on the first one to three days during refeeding, and then returned to the levels of controls. Inconsistently, when restricted rats were allowed *ad libitum* access to food, the food intake increased to twice control levels for 6 days before returning to control levels [45]. One reason for these disparate results may be the length and severity of restriction before refeeding, and animals at a few weeks of severe food restriction will increase food more intake when allowed to eat *ad libitum* [45], [46]. Another reason may be that food intake during refeeding is proportional to the amount of depletion in energy stores caused by food restriction [45], [47]. Here we allowed striped hamsters to restrict to 85% of initial food intake, but fat mass decreased by 56%, indicating that the two explains above might not be the case. It may reflect a special energy strategy in response to food restriction and refeeding in striped hamsters.

In the present study, digestibility did not change in striped hamsters during food restriction and refeeding, indicating that restricted hamsters were not able to enhance their digestive efficiency to extract more energy from digested diet. This suggests that adaptive regulation of energy expenditure is more important than energy intake in the trade-off of the energy strategy in food-restricted animals [10], [40]. The maintenance requirements include the energy exported for RMR and activity. Some food-restricted animals, like MF1 mice [10], deer mice (*Peromyscus*) [48] and chipmunks (*Eutamias minimus*) [49] are reported to decrease RMR and activity to completely compensate for the restricted energy intake, and consequently to prevent weight loss [10]. This is largely different from the results from striped hamsters. Here, we found significant increases in RMR and the time spent on activity in food-restricted hamsters, which was consistent with Syrian hamsters (*Mesocricetus auratus*) and house mice (*Mus musculus*) [49]. This may reflect a different strategy associated with activity for coping with food restriction between different rodent species [50]. An increase in activity in food-restricted animals may indicate an increased effort in foraging, food hoarding or migratory behavior [15], [50]–[52]. Further, an increase in time spent on activity was attenuated in restricted hamsters on day 28, and increased RMR was observed on day 7 but not on day 28, suggesting time-dependent responses to food restriction.

It has been well established that leptin plays a crucial role in the regulations of energy balance [18], [19], [41]. Here, we found significant reductions in serum leptin level in food-restricted hamsters, which was in parallel with the marked decreases in body fat, consistent with the results from other rodents [5], [6], [17]. The body fat loss was 1.3 g in FR-d 28 groups compared with their counterpart controls. Since 1 g adipose tissue contains about 0.8 g lipid (39 kJ/g) and thus contains 31.2 kJ energy [53], [54], 40.6 kJ energy would be mobilized in hamsters during a 4-week's food restriction. On average, the accumulative energy intake of hamsters during the 4-week's food restriction was 1540 kJ, (the accumulative food intake between day 1 and 28 (g)×energy content of the diet (kJ/g)). Thus, the contribution of the body fat loss to the total energy budget would be 2.6%, making us to assume that the fat reduction may induce a lower leptin levels rather than energy provision. Inconsistent with the reductions in leptin level, the time spent on activity increased in food-restricted hamsters. When these hamsters were subjected to a chronic administration of leptin, a significant reduction in activity was observed. Similarly, leptin administration to food-restricted rats, mice and Siberian hamsters attenuated or prevented running wheel activity or food hoarding behavior [15], [55], [56]. These findings may suggest that leptin functioned as a starvation signal to induce an increase in activity levels, making animals to forage, food hoarding or migrate.

Leptin is previously assumed to be an important signal for the switch between fed and fasted states, allowing leptin to function both as a starvation and satiety signal [5], [6], [17], [57], [58]. Here, we also observed significant increases in serum leptin level in striped hamsters during refeeding. These hamsters showed short “compensatory growth” on the first few days during refeeding and recovered body mass and fat mass to the levels of controls, while these animals did also exhibit resistance to overweight relative to their counterparts. An increase in fat storage would enhance the probability of surviving the period of food shortage, but probably simultaneously increases the probability of being killed by a predator [59]. The risks of predation would be a possible interpretation for this resistance to overweight in striped hamsters. Like other rodents [2], [3], [6], [7], [60]], striped hamsters show hyperphagia after being refed *ad libitum*, but it is so short. Leptin supplement attenuated the increase of food intake during refeeding, and leptin was negatively correlated with energy intake in hamsters refed for 7 and 28 days, indicating that leptin presence might attenuate the hyperphagia when food was plentiful, consequently preventing over-weight and also decreasing the risk of predation. In detail, we observed that attenuation of food-intake during refeeding period was transient, and food intake on day 14 was similar in both groups. We also found a lack of leptin effect on time spent on activity on day 12 compared to day 14. Thus a short vs long-term effect of leptin supplement during refeeding period was of interest and needed to be carefully addressed in the further study. In addition to striped hamsters, exogenous leptin completely inhibits food deprivation-induced increased food hoarding and intake in Siberian hamsters [15]. Leptin administration has a similar effect on food intake in rats [61] and mice [60]. These findings may suggest that leptin plays a crucial role in controlling food intake in animals with physiological hyperphagia induced by food restriction and refeeding as that taking place in striped hamsters. Based on the findings of this study, there were two possible explanations of the resistance to overweight or obesity. First, this strain of hamster only showed a transient increase in food intake when food restriction ended, and did not develop hyperphagia. Second, energy expenditure associated with activity and RMR did not decrease in refed hamsters compared with their *ad libitum* fed counterparts. Refed hamsters characterized by the lack of hyperphagia and decreases in energy expenditure were likely reach a new energy balance, consequently resulting in a resistance to overweight or obesity.

In the present study leptin administration to ad libitum hamsters unexpectedly did not significantly affect either food intake or activity behavior. It is unclear why there is a different response to leptin supplement between *ad libitum* and food-restricted hamsters. The roles of leptin are dependent on both circulatory leptin levels and brain leptin transport. Leptin has been shown to be transported into the rodent brain by a saturable process [62], [63]. A possible explanation for this discrepancy is that the transport may be saturated in *ad libitum* hamsters regardless of the exogenous leptin and consequently show a resistance to peripheral leptin injection. Consistently, leptin treatment has a minimal effect on normal humans [63], [64]. In addition, several orexigenic peptides expressed in arcuate hypothalamic neurons including neuropeptide Y (NPY) and agouti-regulated peptide (AgRp), and anorexigenic peptides, e.g., pro-opiomelanocortin (POMC) and cocaine- and amphetamine- regulated transcript (CART) are found to mediate leptin action on energy balance and behavior [63]. A further study on the response of these neuropeptide to exogenous leptin would be needed to explain the discrepancy of the roles of leptin *in ad libitum* hamsters and animals under food restriction and refeeding.

## Conclusion

Striped hamsters showed significant reductions in body mass, body fat content and serum leptin level, and exhibited increases in RMR and activity after being restricted to 85% of initial food intake. After being refed *ad libitum*, hamsters returned body mass, fat mass as well as serum leptin to the levels of controls, showing a “compensatory growth”, rather than overweight. In addition, striped hamsters showed a short hyperphagia on the first few days during refeeding. Leptin supplement decreased activity and attenuated the increase in energy intake. These findings suggest that the decreased leptin level during food shortage perhaps functions as a starvation signal to increase activity behavior, and when food is plentiful the increased serum leptin serves as a satiety signal to prevent activity. Finally, leptin may play a crucial role in controlling food intake and consequently preventing overweight and obesity in animals with physiological hyperphagia caused by food restriction and refeeding.
